# ST6Gal1 is up‐regulated and associated with aberrant IgA1 glycosylation in IgA nephropathy: An integrated analysis of the transcriptome

**DOI:** 10.1111/jcmm.15664

**Published:** 2020-07-17

**Authors:** Youxia Liu, Fanghao Wang, Yaru Zhang, Junya Jia, Tiekun Yan

**Affiliations:** ^1^ Department of Nephrology Tianjin Medical University General Hospital Tianjin China; ^2^ Department of Nephrology Hunan Second People's Hospital Hunan China

**Keywords:** B lymphocyte, galactose‐deficient IgA1, IgA nephropathy, RNA sequencing, ST6Gal1

## Abstract

Galactose‐deficient IgA1 (Gd‐IgA1) plays a crucial role in the development of Immunoglobulin A nephropathy (IgAN), however, the underlying pathogenic mechanisms driving Gd‐IgA1 production in B cells are not well understood. In this study, RNA‐seq analysis identified 337 down‐regulated and 405 up‐regulated genes in B cells from 17 patients with IgAN and 6 healthy controls. Among them, ST6Gal1, which was associated with IgAN in a previous genome‐wide association study (GWAS), was up‐regulated in IgAN and significantly positive correlated with elevated Gd‐IgA1. In addition, we identified increased plasma ST6Gal1 levels in 100 patients with IgAN, which were associated with higher levels of proteinuria, plasma IgA, Gd‐IgA1 levels, greater degrees of systemic complement activation including C3a, Bb, C4d, MAC and a lower proportion classified as C2 grade (crescent proportion ≥25%). Interesting, in vitro, recombinant ST6Gal1 (rST6Gal1) exposure reduced the production of Gd‐IgA1 in cultured peripheral blood mononuclear cells from IgAN patients. rST6Gal1 stimuli also increased expression of *C1GALT1*, which were well‐known proportional to the decrease in galactose deficiency of IgA1. In conclusions, we identified increased plasma ST6Gal1 levels and the association of ST6Gal1 with disease severity of IgAN. Additionally, rST6Gal1 administration in vitro increased expression of *C1GALT1* and reduced the production of Gd‐IgA1.

## 1 INTRODUCTION

Immunoglobulin A nephropathy (IgAN) is the most common glomerulonephritis in the world, with 10%‐20% of patients will progress to end‐stage kidney disease (ESKD) within 10 years after diagnosis.^1^, ^2^ The initial event in the pathogenesis of IgAN is increased synthesis of galactose‐deficient IgA1 (Gd‐IgA1), which lead to IgA1 self‐antibody formation and produce progressive kidney injury.^3^, ^4^ In circulation, B lymphocytes are the major cells for IgA1 production. To date, the cause of production of Gd‐IgA1 in B lymphocyte remains to be determined.

RNA sequencing (RNA‐seq) is a useful tool to study the transcriptome. It can be used as an alternative to microarrays for gene expression analysis, without prior knowledge of the RNA sequence.^5^, ^6^ RNA‐seq provides more accurate data and applications in identifying differently expressed genes between pathological and control samples. Several investigators have sought to identify specific genetic markers associated with the development and progression of IgAN through microarray profiles.^7^, ^8^ However, few studies have specifically described intracellular mechanisms in B lymphocyte associated with IgAN.

To our knowledge, this is the first study to evaluate the global mRNA expression profile in B lymphocyte of IgAN patients, which are directly involved in the disease. In this study, we identified that rST6Gal1 reduce production of Gd‐IgA1 by up‐regulating *C1GALT1* mRNA, suggesting the potential therapeutic value in IgAN.

## 2 MATERIALS AND METHODS

### 2.1 Sample collection

A total of 100 patients with IgAN diagnosed in Tianjin Medical University General Hospital from July 2017 to December 2019, and 50 healthy participants were included in this study. Plasma from all participants was collected. Written informed consent was obtained from each patient and healthy participant. Clinical information and histological grading, including age, gender, 24‐hour urine protein excretion, blood pressure, serum creatinine, total IgA levels, and Oxford classification M (%), E (%), S (%) and T (%), were collected at the time of renal biopsy (Table 1).

**TABLE 1:** The baseline data for patients with IgAN and healthy controls

Characters	Mean ± SD or n (%)	
	IgAN	Healthy controls
Gender (M/F)	55 (55)/45 (45)	23 (46)/27 (54)
Age (mean ± SD, y）	39.35 ± 12.78	36.20 ± 10.08
Serum creatinine (μmol/L)	69.66 ± 50.34	
eGFR (mL/min/1.73 m^2^)	87.46 ± 28.89	
Uric acid (μmol/L)	376.32 ± 115.58	
Bb (μg/mL)	4.41 ± 1.77	
Serum C3a (ng/mL)	1802.04 ± 3088.94	
C4d (μg/mL)	7.50 ± 5.86	
Serum C5b‐9 (ng/mL)	664.08 ± 415.18	
Serum IgA (mg/dL)	335.28 ± 133.41	
Serum IgG (mg/dL)	1092.33 ± 271.38	
Serum IgM (mg/dL)	117.66 ± 59.80	
Serum C3 (mg/dL)	90.75 ± 17.69	
Serum C4 (mg/dL)	23.21 ± 6.89	
Proteinuria (mg/24 h)	1761.07 ± 1608.18	
Urine RBC (/HP)	70.67 ± 214.41	
Oxford classification		
M score (M0/M1)	7 (7)/93 (93)	
E score (E0/E1)	47 (47)/53 (53)	
S score (S0/S1)	60 (60)/40 (40)	
T score (T0/T1/T2)	44 (44)/46 (46)/10 (10)	
C score (C0/C1/C2)	24 (24)/64 (64)/12 (12)	

The study was initially conducted on 47 patients with IgAN and 36 healthy participants. 17 patients (5 with low levels of Gd‐IgA1, 6 moderate levels of Gd‐IgA1 and 6 high levels of Gd‐IgA1) with IgAN and 6 healthy participants were included in the RNA‐seq experiment, 20 participants from each group were used for RNA‐seq validation, and 10 participants from each group were used for the in vitro intervention experiment in PBMCs. The healthy controls were selected on the basis of their demographic characteristics, age and gender overlapped with IgAN group.

### 2.2 B lymphocytes isolation

About 5 mL venous blood sample was taken into ethylenediaminetetraacetic acid (EDTA) anticoagulated tubes. Peripheral blood mononuclear cells (PBMCs) were separated by density‐gradient centrifugation on Ficoll (TBD), sequential washed three times with phosphate‐buffered saline (PBS) and resuspended in PBS + 1% bovine serum albumin (BSA). Peripheral B lymphocytes were isolated using CD19+ magnetic beads (Miltenyi Biotec) according to the manufacturer's instructions.

### 2.3 RNA extraction and RNA deep sequencing

Total cellular RNA was extracted from CD19 positive B lymphocytes using the using a miRNeasy Micro Kit (Qiagen) following the manufacturer's instructions. The RNA concentration was measured using a NanoDrop 2000 spectrophotometer (Thermo Electron Corporation) at 260/280 nm. The NGS libraries were prepared using VAHTS mRNA‐seq v2 Library Prep Kit for Illumina (Vazyme). RNA samples in each group were sent for mRNA deep sequencing on an Illumina HiSeq X sequencing platform with 6GB reads (Illumina). Differential expression gene analysis was performed using R v3.2.2. The log2 transformation was used to obtain the standardized expression values. The *t* test was used to detect differentially expressed genes between patients with IgAN and controls. Significantly up‐regulated genes were defined by as a logarithmic transformed fold‐change (FC) >  0.26 and *P* value ≤.05. Significantly down‐regulated genes were defined by a logFC ≤ 0.26 and *P* value ≤.05. The data discussed in this publication have been deposited in NCBI sequence read archive (SRA) and are accessible through SRA Series accession number PRJNA563895.

### 2.4 Reverse transcription PCR (RT‐PCR)

cDNA was synthesized using total RNA with revert first‐strand cDNA kit according to manufacturer's protocol (Promega). Resulting cDNA was amplified with a 20 µL reaction mixture using SYBR Green PCR Master Mix (Roche) in an Applied Biosystem 7500 Real‐Time PCR System. And the primer pairs of validated genes were listed in Table S1. The fold change between patients and controls was expressed by the 2^−△△CT^ method. The GAPDH gene amplification was used as a reference standard to normalize the target signal.

### 2.5 Plasma ST6Gal1 detection

Plasma ST6Gal1 level was determined by a commercial enzyme‐linked immunosorbent assay (ELISA) kit according to the manufacturer's instruction (Abcam). Plasma samples were diluted 1:20 with diluent. At last, the absorbance was detected at 450 nm with an EL312 Bio‐Kinetics microplate reader (Bio‐TekInstruments).

### 2.6 Assay for IgA and Gd‐IgA1

IgA and Gd‐IgA1 levels in plasma and in cell culture supernatant were detected using a commercial ELISA kit, as previously reported.^9^ Plasma concentration of Gd‐IgA1 was detected according to the manufacturer's instruction (IBL). Plasma samples were diluted with EIA buffer. Diluted plasma and cell culture supernatant were incubated for 60 minutes at room temperature. After washing four times with wash buffer, prepared labelled antibody was added to incubate for 30 minutes. Plate was washed and added 50 μL TMA solution incubation for 30 minutes in dark. Subsequently, the colour reaction was stopped and the absorbance was measured at 450 nm.

### 2.7 Plasma complement component levels

We randomly selected 40 patients with IgAN and detected complement activation products. The levels of human complement components, including C3a, Bb, C4d and C5b‐9 (MAC), were determined according to the manufacturer's specifications by ELISA (Quidel).

### 2.8 Peripheral blood mononuclear cells culture and treatment

Briefly, PBMCs were isolated by density gradient centrifugation and cultured in the RPMI‐1640 medium supplemented with 10% foetal calf serum at 37°C in a humidified 5% CO_2_ incubator in the subsequent steps. In the in vitro experiment, PBMCs were seeded into 24‐well plates and incubated with 0, 100 ng/mL, 200 ng/mL 500 ng/mL human recombinant ST6Gal1 (rST6Gal1, R&D Systems) for 48 hours. The supernatants were collected for detection of IgA1 and Gd‐IgA1 levels. The cells were collected to detect *C1GALT1* mRNA levels.

### 2.9 Statistical analysis

For continuous variables, data with a normal distribution were expressed as the mean ± SD and compared by an unpaired *t* test. For non‐normally distributed variables, data were expressed as the median (first quartile and third quartile) and analysed by the Mann‐Whitney *U* test. Categorical variables were summarized as proportions and were compared by a χ^2^ test. A 2‐tailed *P*‐value <.05 was considered statistically significant. Statistical analysis was performed using SPSS 16.0 software.

## 3 RESULTS

### 3.1 Identification of differentially expressed mRNAs in patients with IgAN and function analysis

We analysed the global gene expression profile in B lymphocyte of 17 patients with IgAN and 6 healthy controls to identify differentially expressed mRNAs. Among 742 dysregulated mRNAs, 337 were down‐regulated and 405 up‐regulated in IgAN. A heat map visualization of the mRNA expression profile was displayed in Figure S1. To analyse the related functions of the dysregulated genes, KEGG pathway analysis was performed. Figure S2 showed the top deregulated enriched pathways in B lymphocyte. The KEGG enrichment pathway analysis of our data revealed that these down‐regulated genes were enriched in herpes simplex virus 1 infection, phospholipase D signalling pathway, autophagy, mTOR signalling pathway and mannose type O‐glycan biosynthesis (Figure S2A); these up‐regulated genes were enriched in ribosome, oxidative phosphorylation, protein processing in endoplasmic reticulum and proteasome (Figure S2B).

### 3.2 Correlation between dysregulated genes expression and Gd‐IgA1 levels

After confirming that IgAN patients had abnormal expression genes, we next investigated whether these genes were correlated with Gd‐IgA1 levels in IgAN patients. The top 10 genes whose expression was correlated with Gd‐IgA1 levels (*R* ^2^ > .28) were listed in Table S2. To validate RNA‐seq results, we chose the top‐ranked 4 genes identified as associated with IgAN and glycosylation according to categories based on their molecular function and biological process. qRT‐PCR was performed for the detection of mRNA levels of *C16orf62*, *GOLGA4*, *BLCAP* and *ST6GAL1* isolated from B cells of an independent set of 20 IgAN patients and 20 healthy controls with the same clinical and demographic characteristics as those in the population used for RNA‐seq experiment. The expression levels of all analysed mRNAs were significantly higher in patients with IgAN, thereby confirming RNA‐seq results (Figure S3).

### 3.3 Patients with IgAN had high levels of ST6Gal1

**FIGURE 1:**
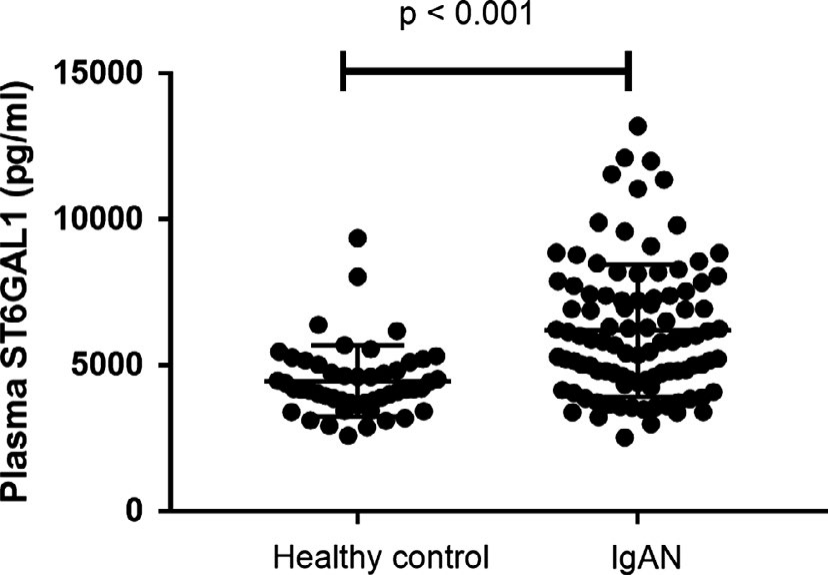
Plasma ST6Gal1 levels in 100 patients with IgAN and 50 healthy controls. In patients with IgAN, the ST6Gal1 levels were significantly higher than those of healthy controls

The mean ST6Gal1 level in plasma in participants with IgAN was 6196 pg/mL, significantly higher than that of healthy controls (4462 pg/mL, *P* < .001) (Figure 1). ST6Gal1 is widely produced by many cell types, such as hepatocytes and B lymphocytes. Therefore, we compared the correlation between the expression of ST6Gal1 in B lymphocytes and plasma. Our results showed that there was a positive correlation between them in IgAN no matter in initial RNA‐seq individuals (*r* = .36, *P* = .09, Figure S4A) or subsequent validation individuals (*r* = .38, *P* = .01, Figure S4B), suggesting that B lymphocytes may contribute to elevated plasma ST6GAL1 levels.

### 3.4 Plasma ST6Gal1 levels correlated with Gd‐IgA1

**FIGURE 2:**
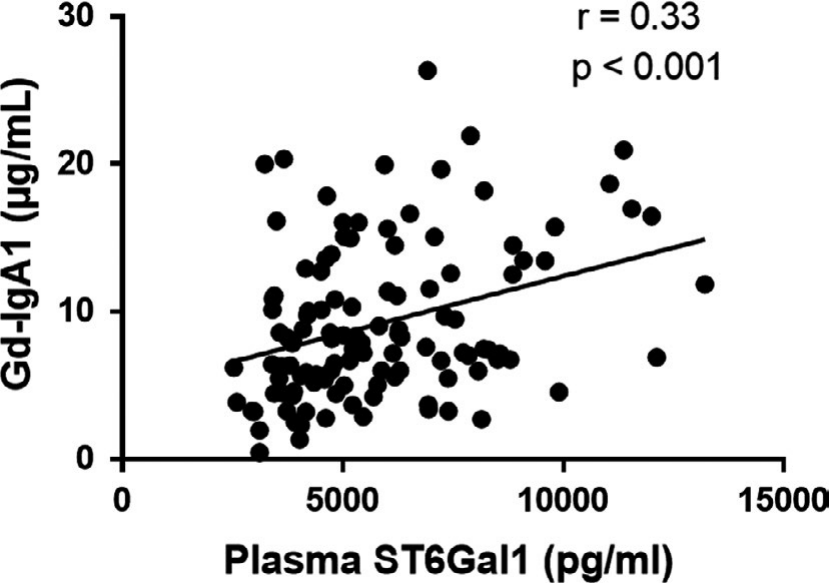
The correlation between ST6Gal1 and Gd‐IgA1 levels. ST6Gal1 levels showed a significantly positive correlation with Gd‐IgA1 levels

After the identification that patients with IgAN had increased plasma ST6Gal1 levels, we investigated whether plasma ST6Gal1 levels were correlated with Gd‐IgA1. There was a strong positive correlation between plasma ST6Gal1 and Gd‐IgA1 levels (*r* = .33, *P* < .001, Figure 2).

### 3.5 Plasma ST6Gal1 levels correlated with severity of IgAN

We further explored the association of plasma ST6Gal1 with clinical findings and pathological lesions in patients with IgAN. We classified patients into 2 groups according to the median value of plasma ST6Gal1 levels (median value: 5825 pg/mL). We found that patients with higher ST6Gal1 levels (>5825 pg/mL) had significantly higher levels of C3a, Bb, C4d and MAC compared with those patients with lower ST6Gal1 levels (≤5825 pg/mL, Table 2). The results also showed higher levels of IgA and proteinuria in higher ST6Gal1 levels group, although the difference was not significant. As for pathological data, we found a lower proportion classified as C2 grade (crescent proportion ≥25%) in patients with higher ST6Gal1 levels.

**TABLE 2:** The baseline data for patients with lower and higher ST6GAL1

Characters	Mean ± SD or n (%)		*P*
	Lower ST6GAL1 (≤5825 pg/mL)	Higher ST6GAL1 (>5825 pg/mL)	
Gender (M/F)	30 (60)/20 (40)	25 (50)/25 (50)	.32
Age (mean ± SD, y)	40.1 ± 12.1	38.6 ± 13.5	.56
Serum creatinine (μmol/L)	69.22 ± 49.08	70.10 ± 52.06	.93
eGFR (mL/min/1.73 m^2^)	86.19 ± 30.77	88.73 ± 27.14	.67
Uric acid (μmol/L)	387.88 ± 126.00	364.76 ± 104.12	.32
Bb (μg/mL)	3.60 ± 0.49	5.21 ± 2.19	＜.01
C3a (ng/mL)	363.24 ± 266.61	3240.84 ± 3870.66	＜.01
C4d (μg/mL)	4.73 ± 1.34	10.27 ± 7.20	＜.01
C5b‐9 (ng/mL)	450.39 ± 52.49	877.76 ± 502.32	＜.01
Serum IgA (mg/dL)	317.91 ± 142.00	352.65 ± 123.20	.05
Serum IgG (mg/dL)	1060.50 ± 246.12	1124.16 ± 293.52	.28
Serum IgM (mg/dL)	117.53 ± 62.89	117.78 ± 57.19	.98
Serum C3 (mg/dL)	89.03 ± 16.15	92.47 ± 19.11	.38
Serum C4 (mg/dL)	22.86 ± 6.79	23.56 ± 5.92	.61
Proteinuria (mg/24 h)	1595.44 ± 1571.92	1926.70 ± 1642.67	.06
Urine RBC (/HP)	65.42 ± 149.78	75.91 ± 265.32	.81
Oxford classification			
M score (M0/M1)	4 (8)/46 (92)	3 (6)/47 (94)	.70
E score (E0/E1)	24 (48)/26 (52)	23 (46)/27 (54)	.84
S score (S0/S1)	26 (52)/24 (48)	34 (68)/16 (32)	.10
T score (T0/T1/T2)	21 (42)/24 (48)/5 (10)	23 (46)/22 (44)/5 (10)	.92
C score (C0/C1/C2)	8 (16)/30 (60)/12 (24)	14 (28)/32 (64)/4 (8)	.05

**FIGURE 3:**
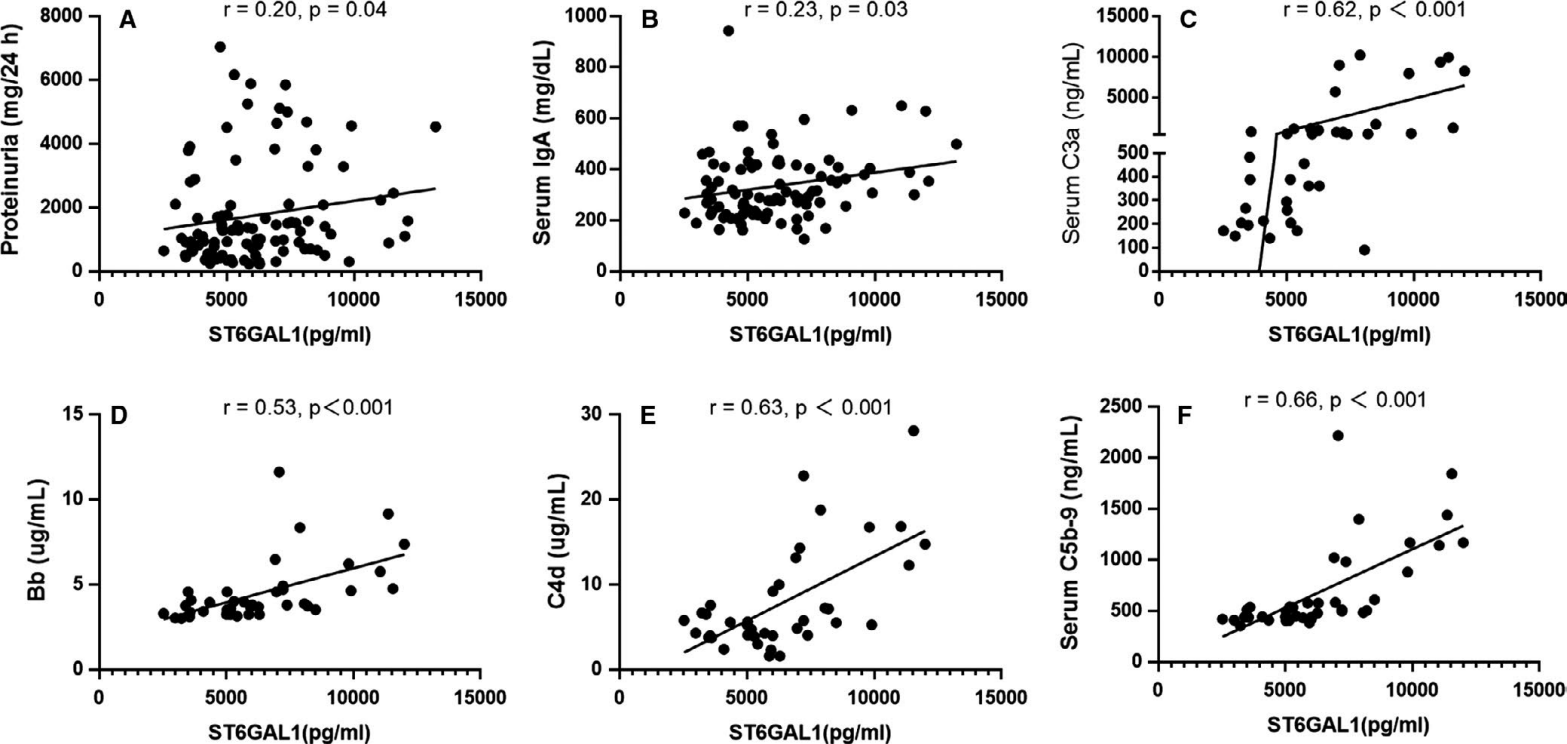
The correlation between ST6Gal1 and clinical parameters. ST6Gal1 levels showed significantly positive correlations with proteinuria (A), total IgA levels (B), C3a (C), Bb (D), C4d (E) and C5b‐9 (F)

In addition, we found that plasma ST6GAL1 levels were positively correlated with proteinuria (*r* = .20, *P* = .04, Figure 3A), total IgA levels (*r* = .23, *P* = .03, Figure 3B), C3a (*r* = .62, *P* < .001, Figure 3C), Bb (*r* = .53, *P* < .001, Figure 3D), C4d (*r* = .63, *P* < .001, Figure 3E) and C5b‐9 (*r* = .66, *P* < .001, Figure 3F). These results indicated that increased plasma ST6GAL1 levels were correlated with severe clinical manifestations in IgAN. No significant association of other clinical manifestations, including urine RBC, eGFR and MEST‐C scores, was observed between patients with higher and lower levels of ST6GAL1 (Table 2).

### 3.6 rST6Gal1 down‐regulated Gd‐IgA1 secretion from PBMCs in a dose‐dependent manner

**FIGURE 4:**
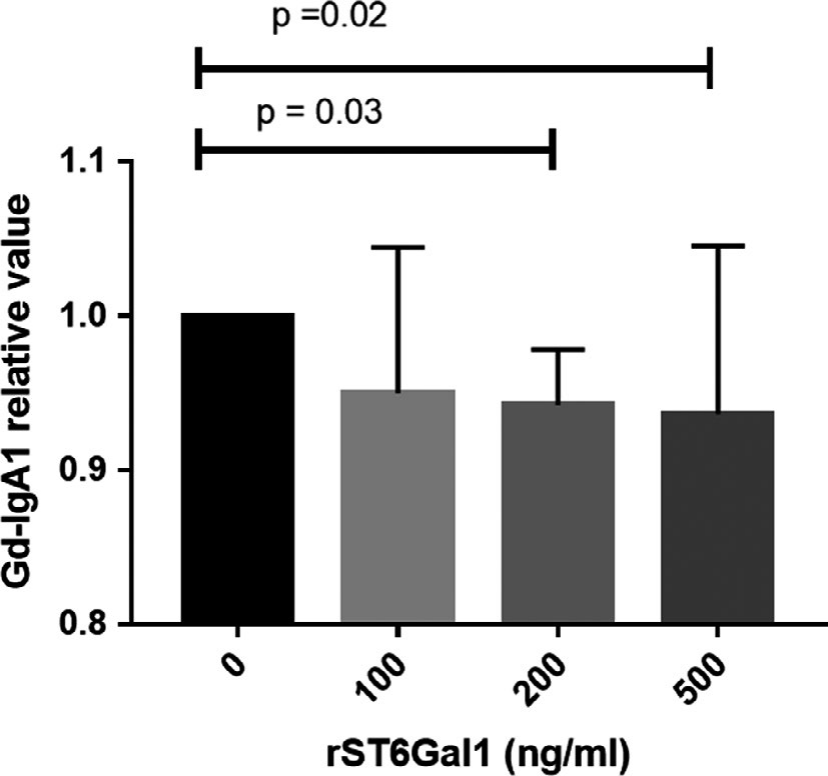
Gd‐IgA1 production after rST6Gal1 stimulation in PBMCs. A scatter plot showed that rST6Gal1 down‐regulated Gd‐IgA1 secretion in PBMCs from patients with IgAN in a dose‐dependent manner

To evaluate whether ST6Gal1 served as the causal factor for increased Gd‐IgA1 in IgAN, we further investigated the influence of rST6Gal1 on the production of Gd‐IgA1 in vitro by different concentration of rST6Gal1 protein. Interesting, we found the addition of rST6Gal1 to the culture medium of PBMCs decreased Gd‐IgA1 production in a dose‐dependent manner (Figure 4). The findings implied a negative regulation of ST6Gal1 in Gd‐IgA1 production in patients with IgAN.

### 3.7 rST6Gal1 up‐regulated *C1GALT1* mRNA expression in PBMCs from IgAN patients in a dose‐dependent manner

**FIGURE 5:**
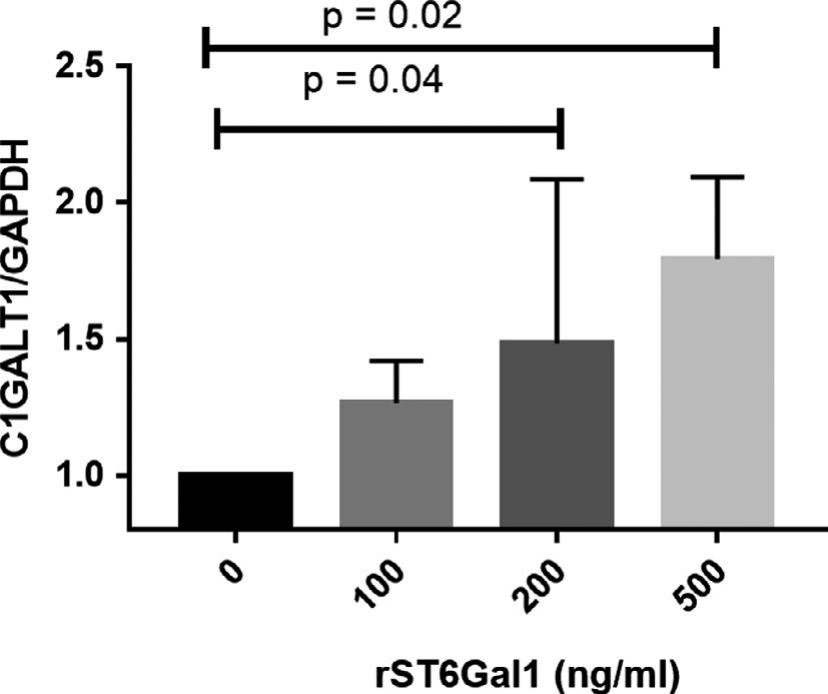
The expression of *C1GALT1* after rST6Gal1 stimulation in PBMCs. rST6Gal1 up‐regulated *C1GALT1* mRNA expression in PBMCs from patients with IgAN in a dose‐dependent manner

We measured the mRNA level of key glycosyltransferases, *C1GALT1* gene, by quantitative RT‐PCR. The result showed that the expression level of *C1GALT1* in PBMCs was up‐regulated as the stimulation concentration of rST6Gal1 increased (Figure 5). The finding fits well with the decreased level of Gd‐IgA1 in cells of IgAN patients stimulated by rST6Gal1.

## 4 DISCUSSION

CD19+ B cells are believed to be the main sources of IgA production and may contribute to the onset and progression of IgAN.^10^, ^11^ In this study, we at first conducted a global RNA sequencing analysis in B cells from patients with IgAN and healthy controls to identify a subset of mRNA transcripts with expression levels correlated with IgAN susceptibility. Furthermore, we cultured human PBMCs to explore molecular mechanism driving Gd‐IgA1 production. By combining these results, we hope to broaden the understanding of pathogenesis of IgAN.

Cell‐specific transcripts are capable of identifying genes associated with clinical phenotypes.^12^, ^13^ Alterations in the functions of genes with cell restricted expression patterns are widely believed to induce specific disease manifestations. From our RNA‐seq analysis, we found 337 down‐regulated and 405 up‐regulated genes in B lymphocytes in patients with IgAN. Pathway analysis revealed that many of these genes were related to autophagy, mTOR signalling pathway, mannose type O‐glycan biosynthesis, ribosome and oxidative phosphorylation et al, which are consistent with previous studies. However, it is difficult to decide which gene can be chosen for a further in‐depth study. On the basis of the pathogenic role of Gd‐IgA1 in the development of IgAN, we investigated whether these genes were correlated with Gd‐IgA1 levels. The results identified the 19 genes whose expression were correlated with Gd‐IgA1 levels with *R* ^2^ > .25. Among these correlated genes, ST6Gal1 aroused our interest. ST6Gal1 encodes ST6 Beta‐Galactoside Alpha‐2,6‐Sialyltransferase 1, mediating the transfer of sialic acid residue with an α‐2,6‐linkage to a terminal galactose.^14^ ST6Gal1 can be found localized in the Golgi apparatus, cell membrane and extracellular region. In a large GWAS comprising a total of 8313 adult patients with IgAN and 19 680 controls of Han Chinese population, Li et al^15^ identified a novel association at ST6Gal1 on 3q27.3 associated with the pathogenesis of IgAN. And risk allele in ST6GAL1 is associated with decreased expression levels of ST6GAL1 in peripheral blood cells and B cells.^15^ However, these correlation results were based on the HapMap data of Europeans and Asians normal healthy population, while not on the IgAN patients. In this study, we found up‐regulated ST6GAL1 expression not only in B cells but also in plasma derived from their IgAN patients. To further validate the result, we also explored ST6Gal1 expression in peripheral blood cell in patients with IgAN and healthy controls from Gene Expression Omnibus (GEO). In consistent with present results, we noticed that the up‐regulated mRNA expression of ST6Gal1 in IgAN patients in PBMCs (GSE 14795, *P* = .04, Table S3). For GSE 58539, ST6Gal1 mRNA level in IgAN was also higher than that of control, although the difference did not reach significance (*P* = .07, Table S3).^16^ Given these evidence, ST6Gal1 appears to be an excellent candidate molecule for further investigation.

We further explored the association of ST6Gal1 on IgAN severity. We observed patients with IgAN who had higher plasma levels of ST6Gal1 presented with higher levels of proteinuria and IgA. Greater degrees of systemic complement activation including C3a, Bb, C4d and MAC were also found in higher ST6Gal1 levels group. However, as for pathological data, we found a higher proportion classified as C2 grade (crescent proportion ≥25%) in patients with lower ST6Gal1 levels.

In order to understand whether higher ST6Gal1 in IgAN is a protective factor or a harmful factor, in vitro experiments were conducted to reveal the causality. The results showed that rST6Gal1 stimuli reduced production of aberrant glycosylated IgA1 molecules in PBMCs derived from the circulation of patients with IgAN. Next, we determined whether rST6Gal1 stimuli affected expression of gene encoding the key enzyme for the glycosylation of IgA1. rST6Gal1 exposure increased expression of *C1GALT1* in IgAN. The increase in *C1GALT1* was proportional to the decrease in galactose deficiency of IgA1. These results indicated that ST6Gal1 was a major molecule reducing the production of Gd‐IgA1 by up‐regulated *C1GALT1* expression, suggesting ST6Gal1 played a protective role in IgAN pathogenesis. Consistent with these results, higher mRNA levels of ST6Gal1 in IgAN patients with normal kidney function (eGFR > 90 mL/min per 1.73 m^2^) than those in patients with mild kidney dysfunction (eGFR 60‐90 mL/min per 1.73 m^2^) was reported.^17^ A study conducted in patients with SLE showed ST6Gal1 levels in B cells were inversely correlated with serum complement 3c (C3c) and C4 level.^18^ Moreover, administration of ST6Gal1‐IgG in mice of Goodpasture disease could convert inflammatory IgG into anti‐inflammatory mediator and reduce inflammatory cell infiltration into the kidney and glomerulosclerosis scoring, effectively attenuating autoimmune disease.^19^ Therefore, we speculated that the ST6Gal1 level in peripheral blood cell was increased by chronic antigen exposure of IgAN. Furthermore, elevated ST6Gal1 inhibited the synthesis of aberrantly glycosylated IgA1 through up‐regulation of *C1GALT1* expression, reducing the amount of auto‐antigen.

## 5 LIMITATION

There are several limitations in the current study. Firstly, peripheral blood mononuclear cells were treated to evaluate causal relationship between ST6Gal1 and Gd‐IgA1 in IgAN. It is difficult to isolate sufficient quantities of B lymphocytes from peripheral blood for in‐depth mechanism study. Secondly, most of the correlation index in the study is about 0.3, it is a weak correlation. Thirdly, the involvement of ST6Gal1 in IgAN should be confirmed by experiments to up‐ or down‐regulate the expression of ST6Gal1. Nevertheless, more studies in widespread populations and B cells are necessary in order to elucidate concrete mechanisms.

## 6 CONCLUSION

Based on transcriptomic data, we identified increased ST6Gal1 levels and the association of ST6Gal1 with disease severity of IgAN. Additionally, rST6Gal1 administration could reduce the production of Gd‐IgA1, which has novel therapeutic potential in the management of IgAN.

## CONFLICT OF INTEREST

The authors declare that they have no competing interests.

## AUTHOR CONTRIBUTIONS

**Youxia Liu: **Data curation (equal); formal analysis (equal); investigation (equal); methodology (equal); visualization (lead); writing – original draft (lead); writing – review & editing (lead). **Fanghao Wang: **Data curation (lead); formal analysis (equal); investigation (equal); methodology (equal); software (equal); visualization (equal). **Yaru Zhang: **Data curation (equal); formal analysis (equal); investigation (equal); methodology (equal). **Tiekun Yan: **Conceptualization (equal); project administration (equal); resources (equal); supervision (equal); validation (equal).** Junya Jia: **Conceptualization (equal); funding acquisition (lead); project administration (lead); resources (equal); supervision (lead); validation (lead).

## ETHICAL APPROVAL

All subjects provided written informed consents. The study protocol was approved by the Institutional Ethical Committee of Tianjin Medical University General Hospital.

## Supporting information

Fig S1

Fig S2

Fig S3

Fig S4

Table S1‐S3

Fig S1‐S4‐Cap

## Data Availability

Raw data used during the current study are available from the corresponding author on reasonable request for non‐commercial use.
