# Supplementary figures and images for: ST6Gal1 is up‐regulated and associated with aberrant IgA1 glycosylation in IgA nephropathy: An integrated analysis of the transcriptome
Source: J Cell Mol Med. 2020 Jul 17;24(18):10493–500. doi: 10.1111/jcmm.15664 (PMC7521275; doi:10.1111/jcmm.15664)

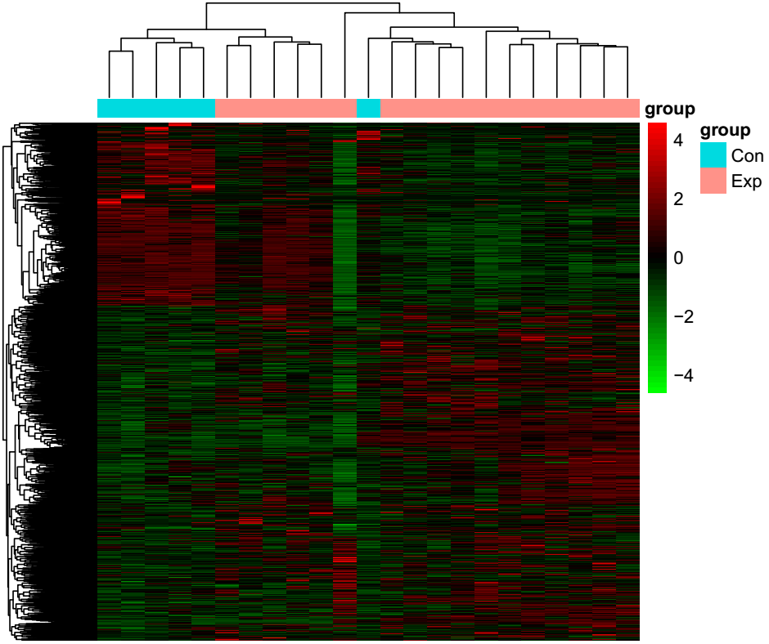

Supplement: Supplementary file 1 — Fig S1 [file JCMM-24-10493-s001.tif]

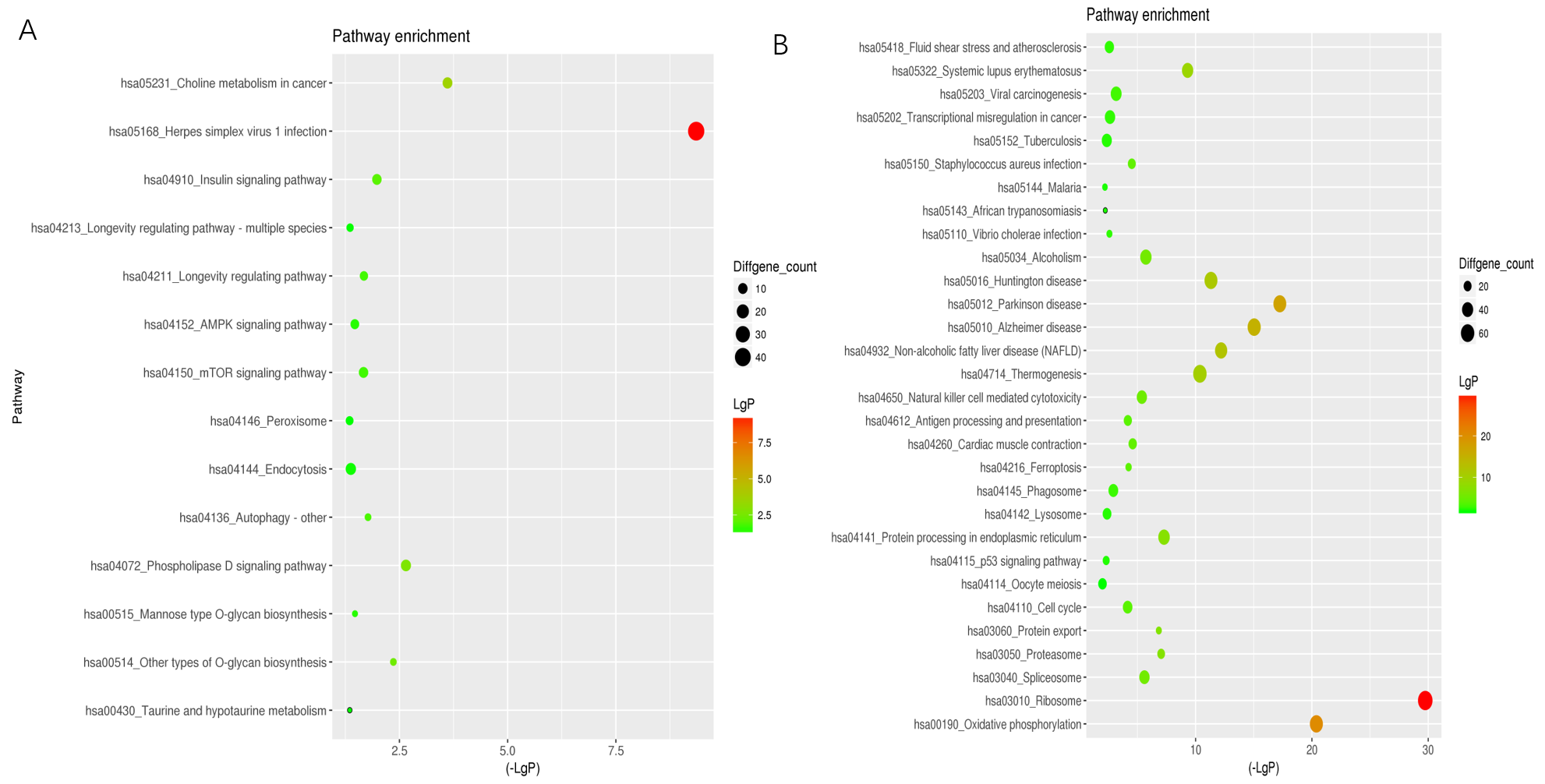

Supplement: Supplementary file 2 — Fig S2 [file JCMM-24-10493-s002.tif]

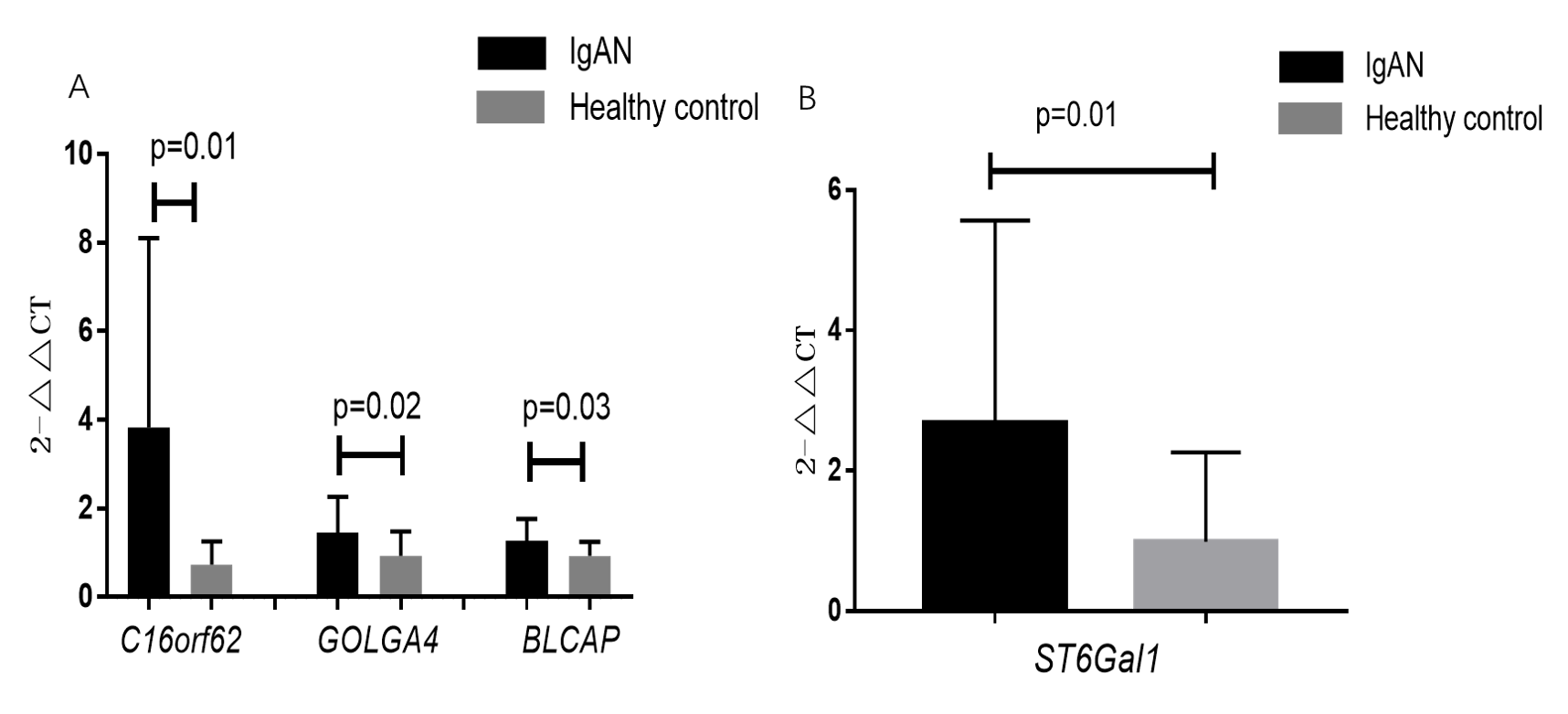

Supplement: Supplementary file 3 — Fig S3 [file JCMM-24-10493-s003.tif]

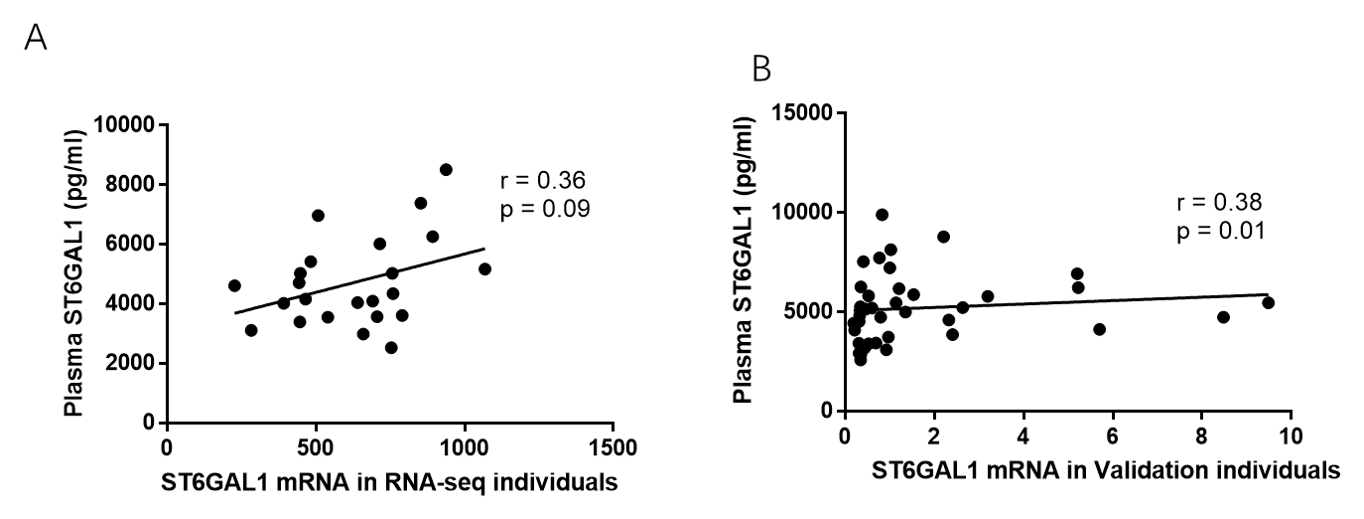

Supplement: Supplementary file 4 — Fig S4 [file JCMM-24-10493-s004.tif]
