# Supplementary material for: ST6Gal1 is up‐regulated and associated with aberrant IgA1 glycosylation in IgA nephropathy: An integrated analysis of the transcriptome
Source: J Cell Mol Med. 2020 Jul 17;24(18):10493–500. doi: 10.1111/jcmm.15664 (PMC7521275; doi:10.1111/jcmm.15664)
Supplement: Supplementary file 5 — Table S1‐S3 [file JCMM-24-10493-s005.docx]

| Supplement Table 1. Primers Used to Amplify the C16orf62, GOLGA4, BLCAP, ST6GAL1, C1GALT1 and GAPDH Genes. | | |
| --- | --- | --- |
| Gene | Forward Primers | Reverse Primers |
| *C16orf62* | CTGCCCCTCACAAGCATGG | TCGGCATGATGCAAATTCAGC |
| *GOLGA4* | ATCCCTGAATCGACTTGACCT | GCAACCGCTGAATCAACTGTT |
| *BLCAP* | GCTCTAGAAGACGGGAGGGGAGGCATTGA | CGGGATCCTTGGGACAGTTGAGAAAGGCA |
| *ST6GAL1* | AACTCTCAGTTGGTTACCACAGA | GGTGCAGCTTACGATAAGTCTT |
| *C1GALT1* | CCTTCCTCTGTGGATCAGCAAT | TTAGGCTGGGTGTCAACCTTT |
| *GAPDH* | GTGGACCTGACCTGCCGTCT | GGAGGAGTGGGTGTCGCTGT |

| Supplement Table 2. The top 10 genes correlated with IgAN and Gd-IgA1 levels with R2 > 0.28. | | | |  |  |
| --- | --- | --- | --- | --- | --- |
| Gene symbol | Full name | log_2_ Fold Change  for IgAN | p for IgAN | R for Gd-IgA1 | p for Gd-IgA1 |
| SNU13 | Small Nuclear Ribonucleoprotein 13 | 1.814151 | 0.0006372 | 0.65451193 | 7.03E-04 |
| GOLGA4 | Golgin A4 | 4.233936 | 0.0355036 | 0.64770308 | 8.33E-04 |
| C16orf62 | VPS35 Endosomal Protein Sorting Factor Like | 7.364683 | 9.531E-05 | 0.57474103 | 0.004123 |
| GLUL | Glutamate-Ammonia Ligase | 3.993506 | 0.0102256 | 0.55597551 | 0.005875 |
| BLCAP | BLCAP Apoptosis Inducing Factor | 3.949151 | 0.0311916 | 0.55479143 | 0.006004 |
| ST6GAL1 | ST6 Beta-Galactoside Alpha-2,6-Sialyltransferase 1 | 0.78989 | 0.0451435 | 0.54642673 | 0.006981 |
| ATF3 | Activating Transcription Factor 3 | 2.212236 | 0.024706 | 0.53792182 | 0.008108 |
| RPS7 | Ribosomal Protein S7 | 1.34834 | 0.0479524 | 0.53098459 | 0.009134 |
| SLU7 | SLU7 Homolog, Splicing Factor | -2.13187 | 0.0003428 | -0.5302225 | 0.009253 |
| OR2T10 | Olfactory Receptor Family 2 Subfamily T Member 10 | -2.6326 | 0.0002388 | -0.5999035 | 0.002479 |

| Supplement Table 3. ST6Gal1 expressions in whole-blood samples from GEO database | | | | | | |
| --- | --- | --- | --- | --- | --- | --- |
| Gene | Experiment E-GEOD-14795 | | | Experiment E-GEOD-58539 | | |
|  | IgAN (n = 12) | Control (n = 8) | p value | IgAN (n = 8) | Control (n = 9) | p value |
| *ST6Gal1* | 85.15 ± 53.90 | 40.98 ± 23.01 | 0.04 | 92.46 ± 4.63 | 88.34 ± 3.98 | 0.06 |
