# Supplementary material for: ST6Gal1 is up‐regulated and associated with aberrant IgA1 glycosylation in IgA nephropathy: An integrated analysis of the transcriptome
Source: J Cell Mol Med. 2020 Jul 17;24(18):10493–500. doi: 10.1111/jcmm.15664 (PMC7521275; doi:10.1111/jcmm.15664)
Supplement: Supplementary file 6 — Fig S1‐S4‐Cap [file JCMM-24-10493-s006.docx]

Supplement Figure 1. Heatmap illustration of the patterns of change in a particular gene across different datasets. Expression levels are represented by red (high) and green (low expression). Samples are from 17 patients with IgAN (red) and 5 healthy controls (blue).

Supplement Figure 2. The top significant KEGG pathways identified in IgAN. The downregulated genes enriched pathways in IgAN (2A) and upregulated genes enriched pathways in IgAN (2B).

Supplement Figure 3. Differential expression validation. Validation of differential expression of *C16orf62* (3A), *GOLGA4* (3A), *BLCAP* (3A) and *ST6Gal1* (3B) in an independent set of B cells from 20 patients with IgAN and 20 healthy controls.

Supplement Figure 4. The correlation between the expression of ST6Gal1 in B lymphocytes and plasma. A positive correlation between them in IgAN no matter in initial RNA-seq individuals (4A) or subsequent validation individuals (4B).
